# Dissociation Pattern in Default-Mode Network Homogeneity in Drug-Naive Bipolar Disorder

**DOI:** 10.3389/fpsyt.2021.699292

**Published:** 2021-08-09

**Authors:** Sujuan Li, Ziwei Teng, Yan Qiu, Pan Pan, Chujun Wu, Kun Jin, Lu Wang, Jindong Chen, Hui Tang, Hui Xiang, Sara Arenas De Leon, Jing Huang, Wenbin Guo, Bolun Wang, Haishan Wu

**Affiliations:** ^1^National Clinical Research Center for Mental Disorders, Department of Psychiatry, China National Technology Institute on Mental Disorders, The Second Xiangya Hospital of Central South University, Changsha, China; ^2^Department of Biochemistry and Molecular Biology, University of New Mexico Health Sciences Center, Albuquerque, NM, United States; ^3^Department of Radiology, The Second Xiangya Hospital of Central South University, Changsha, China

**Keywords:** bipolar disorder, default mode network, network homogeneity, cognitive dysfunction, dyslipidemia

## Abstract

Default mode network (DMN) plays a key role in the pathophysiology of in bipolar disorder (BD). However, the homogeneity of this network in BD is still poorly understood. This study aimed to investigate abnormalities in the NH of the DMN at rest and the correlation between the NH of DMN and clinical variables in patients with BD. Forty drug-naive patients with BD and thirty-seven healthy control subjects participated in the study. Network homogeneity (NH) and independent component analysis (ICA) methods were used for data analysis. Support vector machines (SVM) method was used to analyze NH in different brain regions. Compared with healthy controls, significantly increased NH in the left superior medial prefrontal cortex (MPFC) and decreased NH in the right posterior cingulate cortex (PCC) and bilateral precuneus were found in patients with BD. NH in the right PCC was positively correlated with the verbal fluency test and verbal function total scores. NH in the left superior MPFC was negatively correlated with triglyceride (TG). NH in the right PCC was positively correlated with TG but negatively correlated with high-density lipoprotein cholesterol (HDL-C). NH in the bilateral precuneus was positively correlated with cholesterol and low-density lipoprotein cholesterol (LDL-C). In addition, NH in the left superior MPFC showed high sensitivity (80.00%), specificity (71.43%), and accuracy (75.61%) in the SVM results. These findings contribute new evidence of the participation of the altered NH of the DMN in the pathophysiology of BD.

## Introduction

Bipolar disorder (BD) is a severe mental disorder, mainly characterized by repeated episodes of depression and mania. Depending on its manic level, BD can be further categorized into two types: BD type I (with typical manic episodes) or BD type II (with mild manic episodes) (1). Abnormal functional connections in certain brain areas of patients with BD have been shown with functional magnetic resonance imaging (fMRI) (2). The abnormalities are mainly concentrated in the default mode network (DMN) that comprises a set of brain regions including posterior cingulate gyrus (PCC), precuneus, medial prefrontal cortex (MPFC), bilateral angular gyri (AG), lateral temporal cortex (LTC), and hippocampi (3, 4). Evidence has also shown that DMN plays a crucial role in the pathophysiology of BD (4, 5).

Despite the abnormal functional connectivity (FC) regarding the DMN reported in some BD studies, these findings are still inconsistent (6). In BD type II, both increased and decreased FC associated with the DMN (7) were identified. For example, FC increased between bilateral precuneus and the right Crus I, but decreased between the left MPFC as well as temporal lobe (5) and the cerebellum. Chai et al. (8) and Favre et al. (9) found hyper-connectivity of the MPFC with other cortical regions (e.g., dorsolateral prefrontal cortex) and mesolimbic regions (e.g., amygdala and insula) while Gong et al. (4) observed hypo-connectivity of left PCC to the bilateral MPFC and bilateral precuneus/PCC. It is important to note that the analyzing methods applied in these studies were independent component analysis (ICA) and/or region of interest (ROI) seed-based correlation method. Although these two methods provide important clues for exploring the neurophysiology of BD, both of them have their own drawbacks.It is unclear whether ICA can be used to analyse the difference in components among groups and/or participants (10). As for the ROI seed-based correlation analysis, the placement of the ROI seed is arbitrary in a network (11). New methods that complement ICA and ROI seed-based correlation method to analyze functional neuroimaging data in BD should be considered.

Network homogeneity (NH), a new method for functional neuroimaging data analysis suggested by Uddin et al. (12), is one potential informative approach. It offers an unbiased survey of a distributed network of interest and locates the brain regions that can indicate a disease-related reduction in network coherence. NH refers to the correlation between a specific voxel/brain area and other voxels/brain areas in a local network or in a whole-brain network of interest on the voxel level. In other words, this method mainly studies the consistency of a particular voxel/brain region in the specific network with other voxels/brain regions in the DMN. NH is now widely applied in the research of clinical disease networks such as schizophrenia (13), major depression disorder (MDD) (14), and temporal lobe epilepsy (15). Our research team has already explored the DMN homogeneity in patients with schizophrenia and MDD (14, 16, 17). However, DMN homogeneity in patients with BD has not yet been investigated.

Cognitive dysfunction is one of the hallmarks in patients with BD, with ~40–60% of BD patients suffering different degrees of cognitive deficits, especially in verbal and visual memory as well as executive function (18). Interestingly, cognitive impairment in BD patients was found to be associated with abnormal DMN functional connection. BD patients with less variable connectivity between MPFC and PCC over time showed reduced processing speed and cognitive set-shifting (19). However, the relationship between cognitive dysfunction and abnormal functional connection has never been explored using NH methods.

Different degrees of dyslipidemia, such as increased triglycerides (TG) (20), increased cholesterol (CHOL), low-density lipoprotein cholesterol (LDL-C) (21), or decreased high-density lipoprotein cholesterol (HDL-C) (22) are common in patients with BD. A decrease in HDL-C affects the central nervous system (CNS) and is a risk factor for BD patients (23). A negative correlation between TG and executive function was also reported in a bipolar study after controlling for age, IQ, and the current use of second-generation antipsychotics (20). Decreased serum HDL-C was found to be correlated with cognitive deficits in patients with BD, especially in immediate memory and language domains (24). However, the relationship between dyslipidemia and the DMN in BD has not yet been investigated.

The present study applied the NH method to analyze the changes in the DMN in drug-naive BD patients. The primary aim was to explore the relationship between such changes with cognition and/or blood lipid levels. We hypothesized that abnormalities of homogeneity in the DMN would be present in the NH analysis for patients with BD and that these abnormalities would be associated with cognitive impairment and/or changes in blood lipid level.

## Methods and Materials

### Participants

From March to November 2019, right-handed patients with BD aged 16–45 were recruited from the Second Xiangya Hospital of Central South University, Changsha, Hunan province, China. Patients were diagnosed with BD by two experienced psychiatrists using the Structural Clinical Interview for Diagnostic and Statistical Manual of Mental Disorders, Fifth Edition (DSM-5). It is important to note that these patients had never taken any medication or psychotherapy to treat their BD and that the medical history of BD from onset did not exceed 5 years. The exclusion criteria were as follows: (1) other psychiatric disorders according to the DSM-5; (2) any severe physical diseases such as kidney, cardiovascular, neuropsychiatric disorders or liver diseases; (4) any forms of traumatic brain injury; (5) any history of use of electroconvulsive therapy; (6) pregnancy; and (7) any contraindication(s) for MRI scan. Clinical symptoms of BD were assessed with the Hamilton Depression Rating Scale-17 (HAMD-17) (25), the Young Mania Rating Scale (YMRS) (26) and the Hamilton Anxiety Scale-14 (HAMA-14) (27). Repeatable Battery for the Assessment of Neuropsychological Status (RBANS) (28) was adopted to evaluate the cognitive functions for all the patients.

Right-handed healthy controls were recruited from the local community through advertisements. The age, sex ratio, and years of education of the recruited patients and healthy controls were matched. The healthy controls were screened using the Structured Clinical Interview for DSM-5-Nonpatient Version. The exclusion criteria were: (1) any psychosis symptoms, neurological disease, or substance abuse, (2) first-degree relatives having a history of psychiatric illness.

The study was approved by the ethics committee of the Second Xiangya Hospital of Central South University and was performed in accordance with the Helsinki Declaration. All subjects provided written informed consent after a complete explanation. Parental consent was obtained for participants below 18 years of age.

### Sample Collection

Samples from the patient group were collected to analyze the following parameters: liver and kidney function, blood glucose, TG, CHOL, HDL-C, and LDL-C. Fasting blood samples were taken for biochemical analysis between 7 and 9 a.m. to avoid circadian disruption. The samples were centrifuged at 3,000 rpm for 10 min to separate serum, both of which were later stored at −80°C until analysis.

### Scan Acquisition

Imaging was obtained on a Siemens 3 T scanner. Subjects were asked to remain awake, keep their eyes closed and lie quietly. A foam padding was utilized to limit head motion. The following parameters were used for functional imaging: repetition time/echo time (TR/TE) = 2,000/30 ms, 30 slices, 64 × 64 matrix, 90° flip angle, 24 cm FOV, 4 mm slice thickness, 0.4 mm gap, and 250 volumes (500 s).

### Data Preprocessing

The purpose of data preprocessing is to standardize the brains of different individuals to make them comparable. Besides, data drift caused by head movement and machine instability is eliminated to ensure data quality. In this study, the resting-state fMRI data were preprocessed in the DPABI software (29) (Supplementary Methods).

### Statistical Analyses

The demographics and clinical characteristics of the two groups were analyzed using two-sample *t*-tests, independent *t*-tests, or a chi-square test when necessary.We analyzed NH group differences by voxel-wise cross-subject statistics of the DMN. The ages and mean of the FD were utilized as confounders during the computations. The significance level was set as *p* < 0.05 by using the family wise error (FWE) correction method. Bonferroni approach was used to adjust the *p*-value in the cluster level. Pearson's correlation coefficient was used to analyze the correlations between the NH values and clinical variables in patients with BD.

### Classification Analysis

Support vector machine (SVM), as a form of supervised learning, was adopted for classification. With SVM, classification is still feasible even with a small sample (30). The classification process consisted of two steps: training and testing. First, SVM separated all the subjects with class labels and set up a decision function with its sensitivity and specificity. After the decision function was determined, it was used to predict the classification of new samples. SVM was implemented using the LIBSVM toolbox (LIBSVM software package, http://www.csie.ntu.edu.tw/~cjlin/libsvm/) (31). The LIBSVM software uses the leave-one-out (LOO) method to obtain good sensitivity and specificity through cross-validation. In the study, given a data set containing X (number of cases) samples, X iterations were performed based on the LOO verification. In each iteration, X-1 samples were used to calculate the classifier, and the remaining samples were tested. The kernel type is the default Gaussian kernel in LIBSVM.

## Results

### Demographics and Clinical Characteristics of the Subjects

A total of 43 patients with BD and 37 healthy controls were enrolled in this study. As three patients had excessive head movements, their data were ruled out in the final analysis. Among the remaining 40 patients, 34 patients (85%) experienced depressive episode, 6 (15%) with a mixed episode. No significant differences were observed in age, sex ratios, and years of education between groups (Table 1).

**Table 1:**
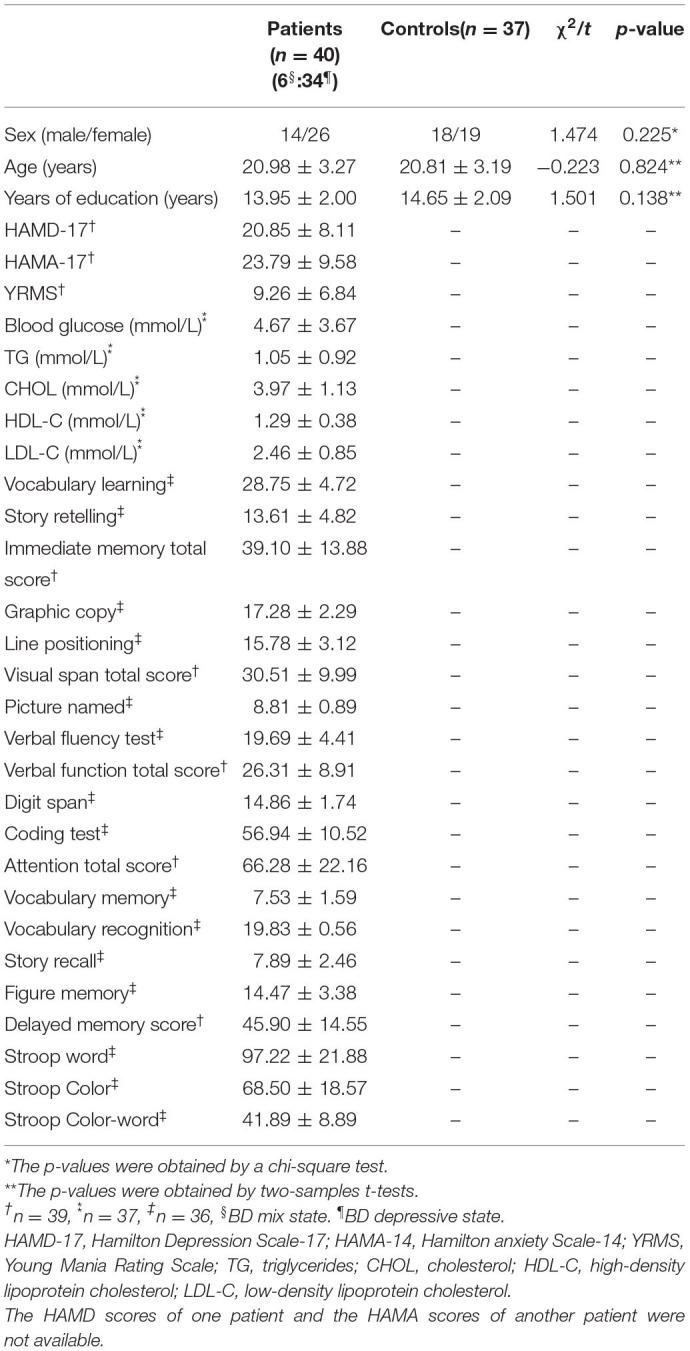
Characteristics of the subjects.

### The DMN Maps Determined by Group ICA

**Figure 1:**
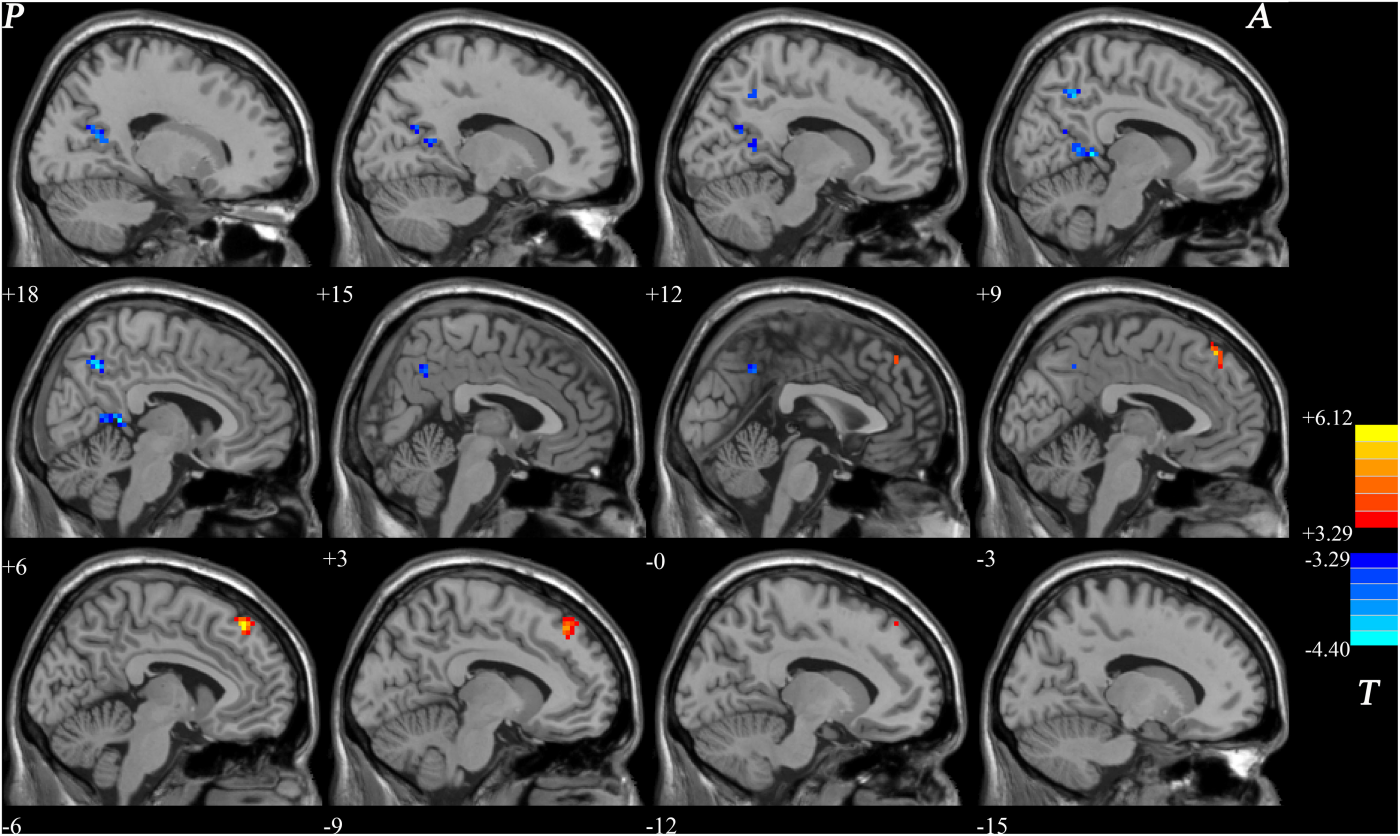
Differences in NH between patients with BD and healthy controls. Increased NH in the left superior MPFC and a decreased NH in the right PCC and bilateral precuneus were observed found in patients with BD. Red and blue denote higher and lower NH respectively and the color bars indicate the *T*-value from two-sample *t*-tests. NH, network homogeneity; BD, bipolar disorder; MPFC, medial prefrontal cortex; PCC, posterior cingulate cortex.

Using a group ICA methods, the DMN mask was selected from the control group. DMN included bilateral MPFC, ventral ACC, PCC, precuneus, lateral temporal cortex, medial, lateral, inferior parietal lobe, and cerebellum Crus I and II (Figure 1). The obtained DMN mask was utilized in the NH analyses.

### NH: Group Differences in the DMN

Significant group difference in NH values in DMN was found *via* voxel-wise cross-subject comparisons within the DMN mask. Compared with the healthy controls, BD patients had lower NH values in the PCC and bilateral precuneus. Significantly higher NH values in the left precuneus and left superior MPFC were observed in the patient group compared with the control group (Figure 1 and Table 2).

**Table 2:** Significant differences in NH values between groups (Figure 1).

**Cluster location**	**Peak (MNI)**			**Number of voxels**	***t*-value**
	***x***	***y***	***z***		
**Patients < controls**					
Right PCC	6	−45	6	49	−4.3953
Bilateral precuneus	6	−60	42	28	−4.2142
**Patients > controls**					
Left superior MPFC	−6	39	51	37	6.1168

*BD, bipolar disorder; NH, network homogeneity; MNI, montreal neurological institute; PCC, posterior cingulate cortex; MPFC, medial prefrontal cortex*.

*The significance level was set at p < 0.05 for multiple comparisons corrected by Gaussian Random Field (GRF) theory (voxel significance: p < 0.001, cluster significance: p < 0.05)*.

### Correlations Between NH and Clinical Variables

**Figure 2:**
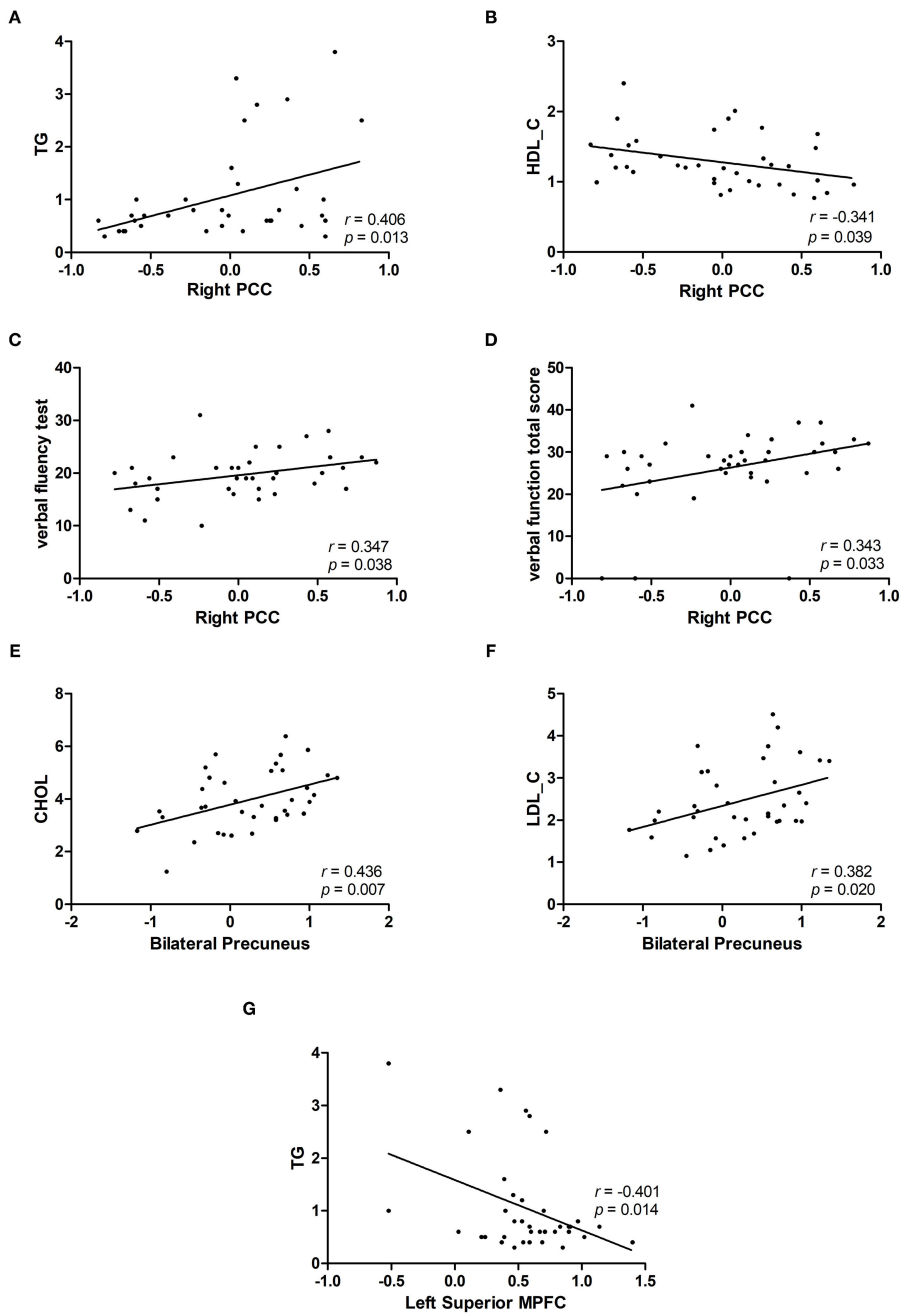
Correlations between abnormal NH values and cognition parameter scores and/or blood lipid level. **(A,C,D)** Positive correlations were observed between the NH values in the right PCC and the TG or verbal fluency test and verbal function total score of RBANS in patients with BD. **(B)** A negative correlation was observed between the NH values in the right PCC and HDL-C in patients with BD. **(E,F)** Positive correlations were observed between the NH values in the bilateral precuneus and CHOL and LDL-C in patients with BD. **(G)** A negative correlation was observed between the NH values in the left superior MPFC and TG in patients with BD. BD, bipolar disorder; NH, network homogeneity; PCC, posterior cingulate cortex; MPFC, medial prefrontal cortex; RBANS, repeatable battery neuropsychological status; TG, triglycerides; CHOL, cholesterol; HDL-C, high-density lipoprotein cholesterol; LDL-C, low-density lipoprotein cholesterol.

We obtained the NH mean values from the PCC, bilateral Precuneus, left superior MPFC and found significant group differences. In patients with BD, we found positive correlations between the NH values in the right PCC and the TG (*r* = 0.406, *p* = 0.013), verbal fluency test (*r* = 0.347, *p* = 0.038), and verbal function total score (*r* = 0.343, *p* = 0.033). A negative correlation was observed between the NH values in the right PCC and HDL-C (*r* = −0.341, *p* = 0.039) in patients with BD. Positive correlations were observed between the NH values in the bilateral precuneus and CHOL(*r* = 0.436, *p* = 0.007) and LDL-C(*r* = 0.382, *p* = 0.020) in patients with BD. A negative correlation was observed between the NH values in the left superior MPFC and the TG (*r* = −0.401, *p* = 0.014) in patients with BD (Figure 2). No significant correlation was found between NH and illness duration, years of education, age, HAMD-17 score, blood lipid level or other cognitive test scores.

### Classification Result

**Figure 3:**
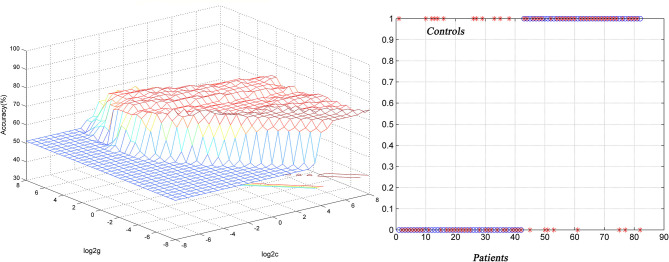
Differentiating the patients from the controls by using the increased NH values in the the left superior MPFC. Visualization of classifications *via* a support vector machine (SVM) by using the NH values in the significantly different regions. Left, result of SVM parameter selection *via* a 3D view; right, classified map of the NH values in the left superior MPFC. NH, network homogeneity; MPFC, medial prefrontal cortex.

SVM results showed that NH in the left superior MPFC had a high sensitivity (75.00%), high specificity (77.78%) and high accuracy (76.47%) (Table 3 and Figure 3).

**Table 3:** Differentiate the patients from the controls by using the NH values of a single region with SVM method.

**Brian region**	**Sensitivity**	**Specificity**	**Accuracy**
Bilateral precuneus	52.50% (21/40)	83.78% (31/37)	68.14% (52/77)
Left superior MPFC	77.57% (31/40)	70.27% (26/37)	73.92% (57/77)
Right PCC	67.50% (27/40)	70.27% (26/37)	68.89% (54/77)

*NH, network homogeneity; SVM, support vector machines; PCC, posterior cingulate cortex; MPFC, medial prefrontal cortex*.

## Discussion

Using the NH method, we provided an unbiased survey of the DMN in first-episode, drug-naive BD at rest. The dissociation pattern of NH in the DMN (increased NH in the left superior MPFC and decreased NH in the right PCC and bilateral precuneus) was identified in the present study. Significant correlations were found between abnormal NH values in the three above-mentioned brain regions and blood lipid level as well as cognitive dysfunction in the patient group. To our knowledge, this is the first study that used NH method to explore changes of the DMN in drug-naive patients with BD and the first to explore the relationship between DMN changes and dyslipidemia. The findings of abnormal NH in the DMN are in line with previous results showing altered FC in the DMN in BD (32, 33), and add a new clue of abnormal network coherence to the role of the DMN in the pathophysiology of BD.

The first significant finding of this study is a dissociation mode in DMN. To our best knowledge, a DMN dissociation in BD was never reported in other studies. We speculate that the dissociation between anterior region (left superior MPFC) and posterior regions (right PCC and bilateral precuneus) could be partially due to the possibility of a functional specialization within the DMN (34). Anterior regions (MPFC, ACC, middle frontal gyrus, and orbitofrontal cortex etc), which has strong connection with limbic areas, participate in representing interoceptive stimuli and processing the emotional self-referential (35). On the contrary, the hippocampus-densely connected posterior regions (PCC, precuneus, AG, etc), by which self-referential stimuli is put within a temporal contextare and linked with past self-referential events, are more involved in retrieving autobiographical memory and coding (36).

MPFC is an important component of the anterior DMN as it plays a key role in the adjustment of cognitive function, emotional behavior and self-reference processes (37, 38). This area is widely connected to emotional marginal regions, executive control regions, and emotional processing regions (39). The recent review supported the evidence that MPFC is also the crucial region of brain reward system in emotional decision-making (40). Any dysfunction in this region, such as the abnormal FC, or increased NH found in the present study, is likely associated with impairment in the modulation of emotional behaviors and cognitive function. It has been confirmed that BD patients with suicidal attempt tend to have an increase in amplitude of low frequency fluctuation (ALFF) in the region of MPFC (41). Another study found that among patients with BD, the inability deactivation of the MPFC during the fMRI task was associated with worse executive function (42). Increased NH in the left superior MPFC may be attributed to the enhancement of self-focus may also drive increase in ruminations as well as negative emotions to oneself (43). Additionally, NH in the left superior MPFC proved its potential as a biomarker to distinguish patients with BD from a healthy population with a high sensitivity, specificity, and accuracy in the SVM analyses in the present study.

As the posterior midline core region of DMN, PCC is mainly associated with autobiographical memory, but it is also involved in maintaining self-awareness, self-guided thinking activities at rest, and the regulation of cognition, emotion, action, and intuition (44). Hypo-activation in the PCC in BD patients implies disturbed integration of emotional information and autobiographical memory related to any intimate relationship (45). Zhong found decreased ALFF in the bilateral precuneus/PCC in the routine and slow-4 frequency bands (46), and a seed-based FC analysis revealed decreased FC between the posterior cerebellum and the precuneus/PCC (46, 47). In addition, structural MRI studies revealed reduced gray matter and white matter volume in the PCC were also found in BD (48, 49). The NH used in this study is a voxel-wise analysis to examine the correlation of a given voxel with all other voxels within a particular network, decreased NH in the right PCC might reflect in the disrupted interaction of right PCC with the whole DMN, resulting in emotion processing difficulties and cognitive dysfunction commonly found in patients with BD which is consistent with previous results (45). This is further supported by the correlation results in the present study. In further correlation analysis, the value of NH in the right PCC was found to be positively correlated with both language fluency and total language function scores in RBANS. A study reported altered regional cerebral blood flow in the bilateral PCC is associated with verbal IQ in mild cognitive impairment (50). In autism spectrum disorders, it was also reported that PCC is related to language abilities (51). Base on these findings, the PCC might be one of the main areas involved in language.

Similar to PCC, the precuneus is also a posterior node of the DMN that is consistently identified in the pathophysiology of BD (52) due to its role in self-referential processing, imagery and autobiographical memory (53). A previous study has exhibited that abnormal FC in precuneus of patients with BD might lead to impaired autobiographical memory (45). Decreased FC of precuneus is found both in BD and MDD. Wang et al. (54) using graph-theory found both the BD and MDD patients showed similarly decreased short-range functional connectivity strength in the bilateral precuneus. Qiu et al. (55) also reported that the fractional ALFF of the precuneus was decreased in both MDD and BD patients with a negative correlation between the fractional ALFF of the precuneus and the cognitive impairment score. In addition to this, one relative structural MRI study has shown that gray matter in precuneus is reduced in patients with BD (56). All of these studies indicated that abnormal activities in the precuneus are likely to be related to affective disorders.

Another important finding of this study is that using a multivariate method, dyslipidemia could be associated with functional connections in the brain. The similarities of schizophrenia and BD in clinical manifestations and etiology have been discussed in many recent works, one of which is a multivariate analysis providing an accurate characterization of the relationship of functional brain activities and psychosis (57). A study based on multimodal assessments reported schizophrenic patients were more strongly affected than BD patients in verbal learning under structural alterations within the hippocampus (58). In this study, we speculate that lipid in the peripheral blood might affect, or be affected by, the brain function in the following ways: (1) From periphery to central nerve system (CNS): the evidence has shown that high concentration of CHOL or TG in the peripheral blood could damage the blood-brain barrier (59), leading to entry of lipid from the peripheral blood to the CNS (60, 61). Consequently, it triggers a series of central inflammation and neurotoxic cascade reactions, resulting in extensive degeneration of neurons (62). (2) From CNS to periphery: Overactivation of hypothalamic-pituitary-adrenal (HPA) axis increases the level of glucocorticoid in the CNS (63)and weakened negative feedback regulation of the HPA axis, giving rise to insulin resistance and metabolic issues such as hyperglycemia or dyslipidemia (64). Overall, despite the absence of direct evidence, dyslipidemia in patients with BD could still be associated with functional changes in the DMN as shown in the present study. This finding may provide some potential evidence for distinguishing schizophrenia from BD in the future.

Apart from the small sample size, some other limitations also need to be considered. Firstly, the control group did not take a cognitive assessment or had the blood lipid checked, which limits comparisons of BD and healthy controls. Secondly, this study is only focused on the study of the DMN, and may ignore the changes in the FC of other brain areas. Finally, patients were not categorized into BD type I or II or mixed state, also did not include other affective disorders, so the presented results were unable to differentiate BD from other affective disorders. The “latent” forms of bipolarity, mixed, and sub-threshold affective syndromes will be collected in the future study. Approximately 85% of patients in this study were in the depressive state so this dissociation pattern in BD could be related with the depressive state as similar DMN dissociation was reported previously (36). Whether DMN dissociation exists in the manic state still remains unknown so DMN in different types of BD are in need of further investigation.

## Conclusion

This study found a dissociative pattern of NH in the DMN (increased NH in the left superior MPFC and decreased NH in the right PCC and bilateral precuneus) in patients with BD. NH abnormalities in the left superior MPFC, right PCC, and bilateral precuneus could be associated with emotional and cognitive dysfunction in the DMN. In addition, NH value in the left superior MPFC can serve as a potential marker to distinguish patients with BD from the healthy. Our study is the first to explore the connection between dyslipidemia and brain function in patients with BD and provide preliminary evidence that dyslipidemia could be related to abnormal DMN connections in BD. In future studies, larger sample size with a longitudinal study design should be considered to explore the possibility of blood lipid level as a marker for identifying brain abnormalities at different stages of BD.

## Data Availability Statement

The original contributions presented in the study are included in the article/Supplementary Material, further inquiries can be directed to the corresponding author/s.

## Ethics Statement

The study was approved by the ethics committee of the Second Xiangya Hospital of Central South University. Written informed consent to participate in this study was provided by the participants' legal guardian/next of kin.

## Author Contributions

HW, BW, and JC conducted and designed the study. SL, YQ, ZT, PP, CW, and LW collected the data. WG and HW analyzed and interpreted the data. HW drafted the manuscript. BW, KJ, SD, JH, and HX provided critical text revision. All authors have contributed to and approve the final manuscript.

## Conflict of Interest

The authors declare that the research was conducted in the absence of any commercial or financial relationships that could be construed as a potential conflict of interest.

## Publisher's Note

All claims expressed in this article are solely those of the authors and do not necessarily represent those of their affiliated organizations, or those of the publisher, the editors and the reviewers. Any product that may be evaluated in this article, or claim that may be made by its manufacturer, is not guaranteed or endorsed by the publisher.
