# Supplementary material for: Dissociation Pattern in Default-Mode Network Homogeneity in Drug-Naive Bipolar Disorder
Source: Front Psychiatry. 2021 Aug 9;12:699292. doi: 10.3389/fpsyt.2021.699292 (PMC8380964; doi:10.3389/fpsyt.2021.699292)
Supplement: Supplementary file 1 [file Data_Sheet_1.docx]

Supplemental Methods

**1.Data preprocessing**

The first step is to standardize the MRI images of each layer obtained at different times to the same time point through Slice Timing. Then, the initial 10 volumes were taken out due to instability of initial MRI signals and for patients to adapt to circumstances. After that, subjects with over 2 mm maximal translation in the x, y, or z-axis and 2°maximal rotation in each axis were excluded by head motion correction. Spatial normalization was applied to normalize the head images of different patients to the same standard space. The imaging data of individuals were spatially normalized to a conventional Montreal Neurological Institute (MNI) echo planar imaging (EPI) template and resampled to 3 mm×3 mm× 3 mm. Finally, by running the band-pass-filtered (0.01–0.08 Hz) and linearly detrending, the effect of high-frequency physiological noise and low-frequency drifts were reduced. Several covariates, including the signal from a ventricular region of interest, the signal from a region centred in the white matter, and Friston-24 head motion parameters obtained via rigid body correction, were removed. The global signal was not removed, as indicated in a previous study. Besides, mean framewise displacement (FD) was used to solve the residual effects of motion as a covariate in group analyses. Scrubbing was also used as an aggressive head motion control strategy (removing time points with FD > 0.2mm).

**2. Default-mode network (DMN) identification**

First, subject- and group-level principal component analyses (PCAs) were performed to reduce the dimensions. The number of independent components (ICs) was assessed with the minimum description length criterion, which was set to 20 in this study. Second, a back-reconstruction strategy was applied to acquire specific ICs based on the group ICs and PCA reduction results. Two DMN components were selected for all participants according to the templates supplied by the GIFT. Third, a statistical map was at the threshold using voxel-wise one-sample t-tests for each component (p < 0.05 for multiple comparisons corrected by Gaussian random field theory, voxel significance: p < 0.001; cluster significance: p < 0.05). Two masks were generated. Finally, the masks were combined to form a DMN mask, which was used as a mask in the following NH analyses.

**3.NH analyses**

NH analyses were performed with an in-house MATLAB script. Correlation coefficients between the time series of a given voxel and the time series of all other voxels within the DMN mask were calculated for each subject. The homogeneity of a voxel was defined as the average correlation coefficient of this voxel. Then, the average correlation coefficients were transformed into z values through Fisher’s r-to-z transformation to generate NH maps. Finally the NH maps were smoothed with the Gaussian kernel of 4mm full-width at half-maximum.
